# Audience Gaze and Felt Social Connection With Individual Performers During Live Dance

**DOI:** 10.1111/nyas.70412

**Published:** 2026-10-19

**Authors:** Albane Arthuis, Sonia Abad‐Hernando, Anastasia Kokkinou, Mirko Febbo, Laura A. Rai, Haeeun Lee, Matthias Sperling, Jamie A. Ward, Daniel C. Richardson, Guido Orgs

**Affiliations:** ^1^ Institute of Cognitive Neuroscience University College London London UK; ^2^ Department of Psychology & Neuroscience, City St George's University of London London UK; ^3^ Department of Computing, Goldsmiths University of London London UK; ^4^ Department of Psychology, Goldsmiths University of London London UK; ^5^ Independent Artist & Choreographer London UK; ^6^ Siobhan Davies Studios London UK; ^7^ Department of Experimental Psychology University College London London UK; ^8^ Institute of Sport, Exercise and Health University College London London UK

**Keywords:** collective attention, collective engagement, dance, gaze, mobile EEG, social connection

## Abstract

In everyday social interactions, our looking behavior reflects our relationships with others, but the role that social perception plays for engagement with the performing arts is understudied. We commissioned the durational, community‐focused, 16‐h dance performance *How Shall We Begin Again?*, comprising a continuous relay of individual improvised performances by 50 people over 2 consecutive days. During the performance, we recorded eye movements and mobile EEG from 88 audience members who entered and exited the performance space freely. Audience members reported on their experience of individual performers immediately after leaving the performance space. Gaze toward people in the scene showed a significant indirect association with felt social connection through self‐reported focus on individual performers; the overall gaze–connection association, however, was not statistically significant. Individual differences in mind wandering, dance experience, and empathy were associated with gaze and engagement measures, but not with felt social connection. Finally, EEG alpha power decreased and EEG delta/gamma power increased when dance was accompanied by music. Together, these data suggest that engagement with dance depends not only on what is being performed (dance/choreography/music) but also on who is performing, highlighting that dance is an inherently embodied art form that can foster social connections between people.

## Graphical abstract

We combined mobile EEG, eye‐tracking, and self‐report to study social influences on audience engagement during a 16‐h live, community‐focused dance performance involving 50 performers and 88 participants. Audience engagement was associated with felt social connection to individual performers, mediated by self‐reported attentional focus but unrelated to how well audience members knew individual performers beforehand. EEG alpha power was reduced when dance was paired with music, while delta/gamma power increased, perhaps reflecting context‐dependent modulation of arousal.

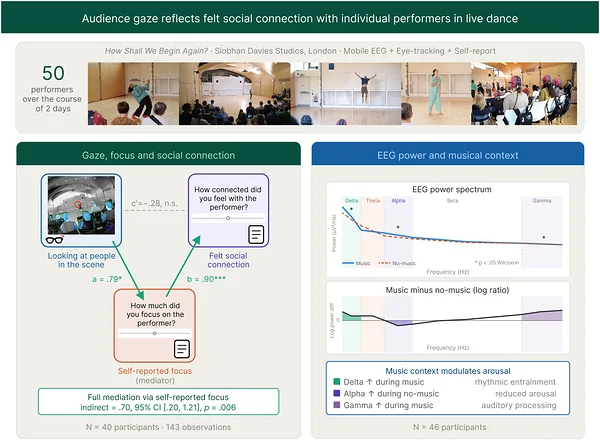

## 1 Introduction

Across cultures, performing arts are shared experiences that foster bonding and belonging [1, 2, 3]. Studying live performance in cognitive neuroscience is challenging because prevailing analyses depend on many experimental trials and are often limited to pairwise social interactions [4, 5]. Dance, music, and cinema performances are physically live [6] because they happen only once in the exact same way and are socially live [7, 8] because they are typically experienced in groups [9].

In a recent mobile electroencephalogram (EEG) study, Rai et al. [8] showed that audience delta‐band synchrony tracks engagement during live dance, and that EEG alpha power differs when watching together versus alone. Interestingly, direct social interactions between performers and spectators in which performers directly looked at individual audience members (breaking the fourth wall) were associated with moments of highest brain synchrony among audience members.

In this study, we explore whether audience engagement, gaze, and mobile EEG measures are associated with felt social connection to dance performers during live dance. We commissioned the durational, community‐focused dance performance *How Shall We Begin Again?*, initiated by director, choreographer, and performer Jo Fong. While some dance performances might be primarily aimed at entertaining audiences, *How Shall We Begin Again?* envisions building a sense of community through participating in a live performance event as a performer, spectator, or both. Over 2 days, 50 performers danced individually for about 25 min while audience members entered and left the performance space freely. The durational and spontaneous structure of the performance deliberately amplifies both physical and social liveness and raises two research questions: (1) to what extent can social connectedness be generated within the context of a live event; and (2) is felt social connectedness with performers associated with audience engagement.

We conceptualize audience engagement as the sustained, multidimensional investment of a spectator in a performance, encompassing both attentional and affective dimensions [4, 10]. We distinguish three components: first, attention refers to the selective allocation of perceptual resources toward a specific stimulus [11], in our case, an individual performer. We measure attention allocation behaviorally indirectly as gaze direction and directly as self‐reported subjective focus. Second, arousal refers to a physiological state of alertness and excitation [12, 13], indexed here by EEG alpha power. Third, social connection refers to the subjective feeling of relatedness to another person [14], indexed here by a single post‐performance rating of felt social connection. Together, gaze, self‐reported focus, EEG power, and felt social connection serve as complementary windows onto audience engagement, each capturing a different dimension of the spectator's investment in the performance.

### 1.1 Social Connection and Gaze

The subjective experience of being with others has a direct impact on how we perceive and engage with the performing arts. In live theater, collective effervescence, the sense of shared emotional experiences in a group, mediates individual enjoyment of the show [15], suggesting that felt connection during performances is not reducible to a purely dyadic audience–performer relationship. Indeed, merely watching videos with others can amplify emotions [16]. Subjective feelings of relating to, or belonging with, others are typically measured via self‐report [14]. When investigating social connection perception in various contexts, some studies have used real‐time sampling of thoughts and feelings [17], but these measures interfere with the experience of the temporal arts, potentially reducing ecological validity [18]. In the present study, we, therefore, use retrospective self‐report measures to capture spectators’ engagement and felt social connection with individual performers, acknowledging that this approach reflects remembered and potentially biased rather than instantaneous in‐the‐moment experience.

Gaze is shaped by social context [19, 20]: gaze timing and focus change depending on whether something is viewed alone or together with others [21, 22]. Generally, eye movements are reliable measures of how well people understand each other in conversation and how connected to others they feel [23]. Gaze thus serves not only as a marker of attention allocation but also as a signal of social interest and relatedness: people look more at those they feel connected to, and looking itself may reinforce felt connection [24, 25]. Moreover, joint attention, that is, two or more people simply attending to the same object/person/location, can increase bonding among romantic partners or among strangers.

Wearable eye‐tracking now enables measuring gaze in dynamic and complex social settings [26], such as live performances. In this study, we use looking behavior as a continuous indirect measure of audience engagement. We predicted that looking at performers/people in the scene is associated with higher felt social connection (H1) and self‐reported attentional focus on the performer (H2).

### 1.2 EEG and Audience Engagement With Music and Dance

Physiological synchrony can index social connection [27], with attentional processes often playing a key mediating role. Within individual brains, alpha power (≈8–13 Hz) attenuates with visual stimulation and with eyes open versus closed [28], varies with eye position, and tracks arousal and attentional demands [12, 13, 29]. Reduced alpha power has also been observed when two people perform a joint attention task [30]. Many forms of dance integrate information across at least two senses, vision and sound, that together generate a multisensory experience over time [31, 32]. In keeping with conventional definitions of dance as movement‐to‐music, people typically prefer dance with music to dance without music [33, 34, 35]. Howlin et al. [33] showed that when people watch a recorded dance performance, movement synchrony among performers predicts electrodermal activity only when movement was paired with sound, but not when the video was watched in silence. Thus, we predicted that arousal as indexed by EEG alpha power might equally depend on the presence of music (H3).

### 1.3 Hypotheses

Our primary hypothesis was that felt social connection with an individual performer is positively associated with gaze toward that performer (H1), mediated by self‐reported attentional focus (H2). Moreover, in keeping with the artistic focus of the show on fostering community between performers and spectators, we predicted that felt connection would arise during the performance itself, rather than being solely determined by prior familiarity with the performer or by stable individual traits such as empathy or prior dance experience. Finally, we predicted that alpha power would be lower (H3) during performance sections with music relative to performance sections without music, reflecting greater spectator arousal when dance is accompanied by music.

## 2 Methods

### 2.1 Performance

*How Shall We Begin Again?* is a dance performance initiated by artist Jo Fong and created and performed by 50 people. The performance took place at Siobhan Davies Studios, London, on November 12 and 13, 2022. The performance took the shape of a live installation with a total duration of 16 h, running continuously for 8 h per day on 2 consecutive days and consisted primarily of an ongoing relay of approximately 25‐min‐long individual improvised performances (or *live investigations*, as Fong terms them) by each of the 50 performers in turn. These individual performances were interspersed at regular intervals with four shorter group sections per day where all performers participate together.^1^ Each performer was invited to contribute 12 pieces of music, of which three were randomly selected and played for them during their individual performance, interrupted by a shorter (mean = 100 s) first and longer second (mean = 252 s) pause. This builds an ongoing alternation between moments with and without music into the performance structure. A fluid process of transition from one live investigation to the next was created by the next performer quietly entering the space during the third track of the current performer, and the current performer exiting when their third track ended, immediately followed by the beginning of the next performer's first track. Each person's live investigation was also accompanied by its own title, chosen by the performer, and projected onto the back wall of the performance space.

While the live investigations were spontaneously generated performances, they drew on tools for improvisation developed in a series of preparatory workshops led by Fong and an artistic support team. The 50 performers were people with a range of different ethnicities, bodies, ages, genders, and a wide range of prior experience of performing. Fong's invitation and challenge to all of the performers was to take the time and space of their live investigation as one where they gave themselves permission to be making themselves at home, while giving the audience permission to witness them in that live situation. Fong describes *How Shall We Begin Again?* as “a creative action; between portraiture, protest and something like a prayer. Just a moment, to be in the moment.” The longstanding focus of Fong's artistic research is on activating the social potentials that live performance holds for and between performers and audience members. *How Shall We Begin Again?* aims to foster community, inclusivity, connection, and belonging among all participants in the show. These artistic intentions of “making the spaces between people available” to social connection directly inform all aspects of Fong's approach to defining the parameters of the performance and the hypotheses of our analyses. Photo, video documentation, and further information on the artistic research are available at www.neurolive.info/performance‐2. For self‐descriptions of the performers and the titles of the individual live investigations, see Tables S1 and S2.

Audience members were invited to book a ticket for an arrival time but could stay as long as they liked. Audience members were able to select between options to book tickets either with or without participation in EEG and eye movement data collection. Figure 1 shows the performance timeline, including individual and group performances, sections with and without music, and the number of EEG and eye movement participants for the entire duration of the show.

**FIGURE 1:**
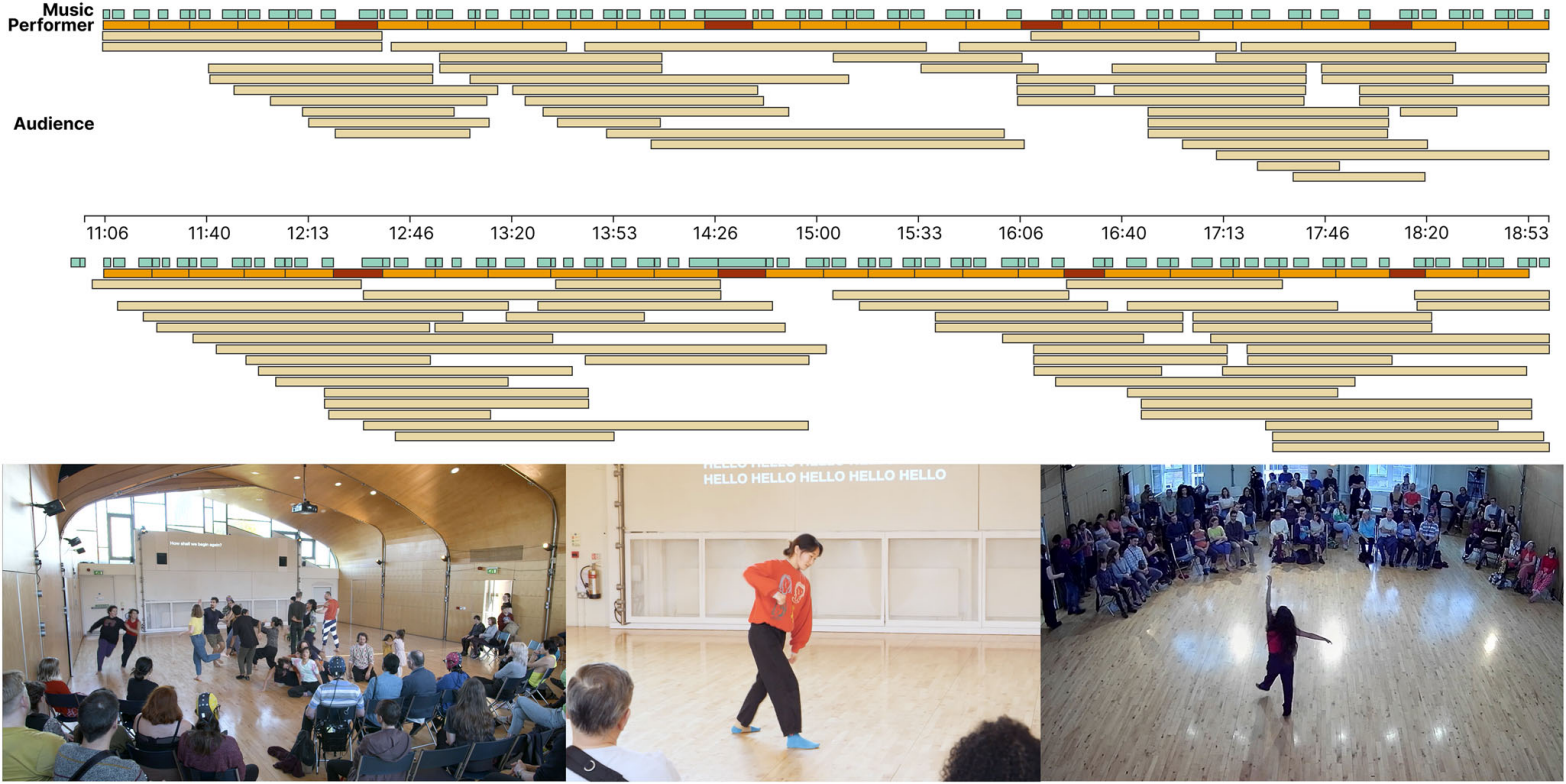
Timeline of participants and performers present in the performance space with pictures of the performance. Top panels: Performance timeline for day 1 (upper panel) and day 2 (lower panel) of the performance, spanning 11:06 to 18:53 each day. The *x*‐axis represents clock time. The top row of each panel shows the structure of the performance: green bars indicate music sections and the spaces between these bars indicate no‐music sections; orange bars indicate individual performances and red darker bars indicate group performances. Each labeled horizontal beige bar represents one EEG/eye‐tracking participant, showing the time window during which they were present in the performance space. Bottom panels: Photographs of the performance space. Left: A group section with multiple performers taken from behind audience members. Center: An individual performance with a performer and their projected title visible on the wall behind, taken from the front row of the audience seating area. Right: An individual performance taken from the stage, audience members visible, sat in a semi‐circle facing the performer. Audience members entered and left the performance space freely during music sections.

### 2.2 Participants

Some audience members were equipped with mobile EEG and eye‐trackers and/or completed questionnaires before and after the show. One hundred and sixty‐five participants completed the pre‐performance questionnaire and 164 completed the post‐performance questionnaire. However, only 108 participants completed both questionnaires. Eighty‐three audience members were equipped with mobile EEG and eye‐trackers. For the analyses of eye movements, 45 participants from this group completed both questionnaires and had clean data. A majority of these 45 participants were female (60%), white (71.11%), and went to university (77.77%); the mean age of participants was 34.7 years (Table S3). After EEG pre‐processing and artifact rejection, 46 out of 83 audience members were included in the power spectral analysis. For EEG post‐performance questionnaire correlations, 34 participants had both clean EEG and complete post‐performance ratings. For EEG‐gaze correlations, 40 participants had both clean EEG and eye‐tracking data. All analyses' sample size and participants' exclusions steps are detailed in Table S4.

All research participants provided informed consent, and the research study received ethical approval from the local ethics committee at Goldsmiths University of London and University College London (protocol number ICN‐VW‐03‐092024A); see Table S3 for research participant demographics.

### 2.3 Pre‐ and Post‐Performance Questionnaires

All questionnaires were implemented using the Gorilla platform [36]. Participants answered online, prior to arrival, or on tablets at the venue for the pre‐performance questionnaire and on tablets at the venue for the post‐performance questionnaire.

#### 2.3.1 Pre‐Performance Questionnaire

The pre‐performance questionnaire contained questions about participants’ demographics (age, gender, ethnicity, and educational level) and four different scales to measure trait empathy (adapted from Batchelder et al. [37]), personality traits using the Big Five 10 item index [38], participatory (PDE) and observational (ODE) dance experience using the Gold‐DSI index [39], and proneness to mind wander, spontaneously or deliberately [40]. Scales were scored according to the original publications’ indications.

#### 2.3.2 Post‐Performance Questionnaire

The post‐performance questionnaire contained the 25 titles of individual improvised performances of each day. Participants were asked to select the ones that they attended (Figure 2). After selecting one of the individual improvised performances, the participants were asked to answer five questions for each of the performers they reported seeing. The performer's visual self‐description was included at the top of the screen to support memory for individual performers. Participants rated on a scale from 0 to 100: “How connected did you feel with the performer?”, “How much did you focus on the performer?”, “How well did you know this person beforehand?”. Participants also completed two open‐ended questions asking them to “Tell us what happened” and “Tell us how you felt” (Figure 2).

**FIGURE 2:**
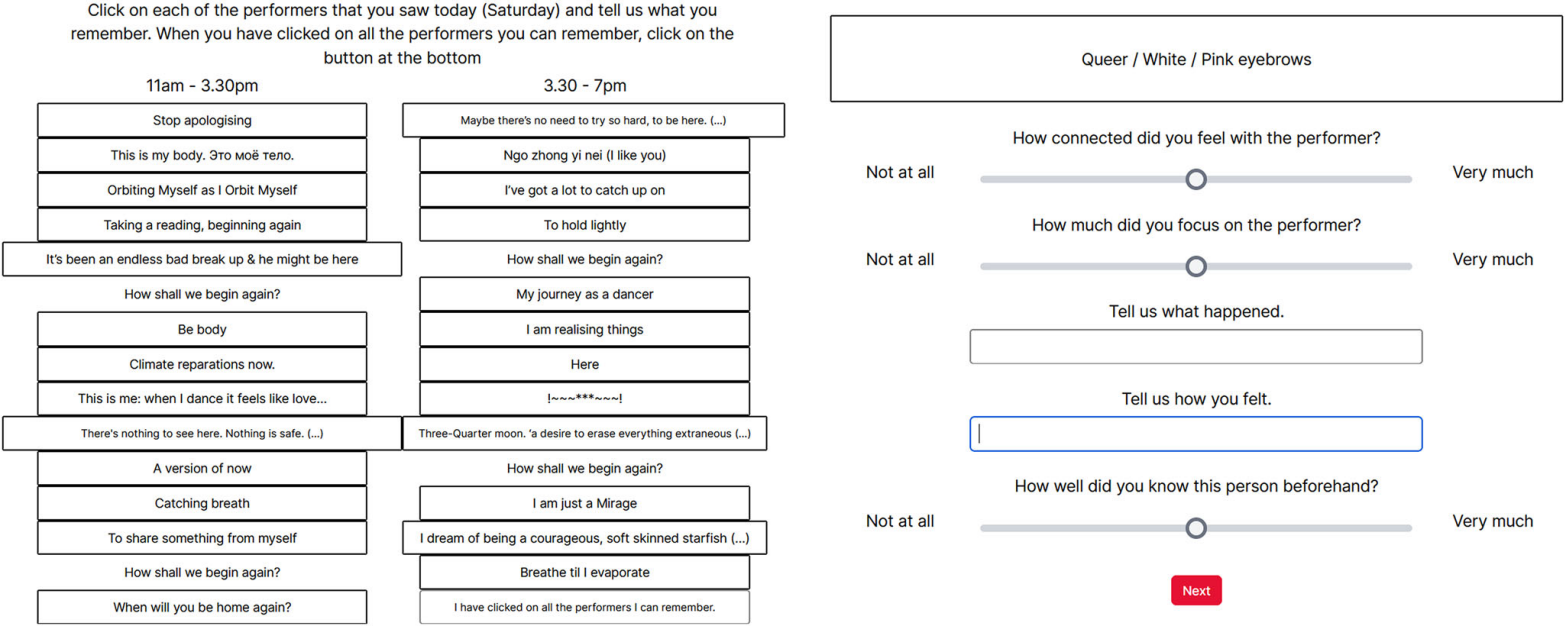
Post‐performance questionnaire screens. Left panel: The performance selection screen, shown separately for the morning session (11 am–3:30 pm, left column) and afternoon session (3:30–7 pm, right column) of 1 day. Each box displayed the title of an individual performance as chosen by the performer. Participants were instructed to click on each of the performances they could remember attending, one by one. Right panel: The individual performance rating screen, which appeared after a participant clicked on a performance title. The performer's visual self‐description appeared at the top (e.g., “Queer/White/Pink eyebrows”). Below, participants rated three items on visual scales from “Not at all” to “Very much”: felt social connection with the performer (“How connected did you feel with the performer?”), self‐reported attentional focus (“How much did you focus on the performer?”), and prior familiarity (“How well did you know this person beforehand?”). Two open‐ended text fields asked participants to describe what happened and how they felt during the performance. Participants navigated between performances using the “Next” button.

The ratings were kept as answered, and the two open questions were analyzed as measures of engagement based on the word count and the positive/negative valence of the words used. To do so, we used the R package of the NRC Word‐Emotion Association Lexicon that performs a sentiment analysis based on the lexicon used by the participant [41].

### 2.4 Materials—Eye‐Trackers

Eye movements were recorded using PupilLabs Invisible eye‐trackers [42]. These devices recorded a video of what is in front of the participant using a camera on the right arm of the glasses and recorded the participants’ eye activity using two small cameras inside the glasses. From this, they computed participants’ blinking activity and fixation points [43]. For this study, we used the video recording (192 × 192 pixels) from each audience member's device and participants’ fixation points for each timestamp. Gaze data were recorded at 200 Hz.

### 2.5 Materials—Mobile EEG

Mobile EEG data were recorded with the ANT eego sports system (32 Ag/AgCl channel wet electrodes on Waveguard caps) [44]. Electrodes were located according to the 10/10 international electrode placement system [45]. Conductive gel (OneStep Cleargel) was applied in the electrode holders using blunt‐tip syringes. During EEG preparation, electrode impedances were set below 20 kΩ. EEG recordings were sampled at 500 Hz, with CPz as the online reference channel. Data were acquired locally with eego system acquisition software on Windows Surface Pro tablets connected to each amplifier. Following data acquisition, the raw EEG data were exported to .cnt format and subsequently converted to .set format for preprocessing in EEGLAB [46] using MATLAB.

### 2.6 Procedure

*How Shall We Begin Again?* was advertised as both a live performance and a scientific experiment. Tickets could be purchased online, on the venue website (https://www.siobhandavies.com/events/neurolive‐begin‐again/). Participants selected either a “Performance Ticket” or a “Performance Ticket + EEG Participation” option. Those selecting a “Performance Ticket” attended the performance and optionally completed pre‐ and post‐performance questionnaires. Those selecting a “Performance Ticket + EEG Participation” were invited to complete questionnaires and took part in EEG and eye‐tracking data collection; they were provided with a video explaining EEG and eye movement measurements and preparation (https://www.youtube.com/watch?v=wneN1C2U92s&t=18s). The performance took place between 11 am and 7 pm each day. Audience members booked an arrival time slot but stayed for as long as they wished. To manage audience flow and equipment preparation, tickets were available for timed entry every 30 min. A few days prior to the performance date, individuals who had booked either ticket option were contacted via email and invited to provide informed consent and complete the pre‐performance questionnaire online. Upon arrival, “Performance Ticket + EEG Participation” ticket holders who had not completed the pre‐performance questionnaire online were provided with a tablet to do so on‐site during EEG setup.

Participants were equipped with mobile EEG [44] and eye‐trackers [42] in a separate room (Research Studio). Eye‐trackers were calibrated by having participants fixate consecutively on two speakers positioned in the upper corners of a wall, at approximately 5 m from the participant. Following device set‐up, participants were guided to the Roof Studio, where the performance was taking place. A dedicated research assistant ensured that people entered and exited the performance space only when music was playing. Inside the studio, participants were free to choose any seat among four rows of seats, arranged in a semi‐circle facing the performance stage, with the back row slightly elevated. The spatial design served to minimize distractions related to people leaving and entering the space (see Figure 1, bottom panel). After leaving the performance space, EEG participants completed the post‐performance questionnaire on a tablet computer or laptop in a separate room in the building.

### 2.7 Annotation and Synchronization

Collecting data from multiple people and sensors presented several challenges, including synchronization across individuals and devices (eye‐trackers and EEG). Ahead of the performance, we developed custom software employing multiple communication protocols, including Lab Streaming Layer (LSL) for EEG and API calls with time echo protocol for eye‐tracking, to facilitate temporal alignment between the two time series [47].

Eye movement and EEG data were recorded to separate devices, and the durational nature of the performance that allowed people to attend the performance for as long as they liked, did not allow us to use a single external signal to provide a ground truth for device synchronization, such as stage lighting [8]. Therefore, EEG and eye movement data cannot easily be mapped onto a single timeline with millisecond accuracy. We, therefore, limit our analysis to summative measures (i.e., percentage of overall looking time or mean alpha and delta power) for sections with or without music and complete individual performances and do not combine eye movement and EEG data on a single timeline.

Eye‐tracker and EEG synchronization across participants was achieved through multiple stages. First, performance annotations were generated through the custom software interface running on a laptop and transmitted simultaneously to both the eye‐tracking and EEG systems. These included automated markers for song onset and offset retrieved via the Spotify API, a heartbeat synchronization signal transmitted every 10 s, and manually entered performance‐related comments logged in real time by a member of the research team (M.F.) (e.g., beginning and end of solo and group sections, laughter, or clapping). Second, a synchronization file was constructed aggregating all sent and received timestamps across devices, allowing comparison of timing across participants. Third, EEG latencies were manually corrected (A.A. and S.A.‐H.) to align each recording to the onset and offset of songs and no‐music sections, ensuring precise temporal alignment of all EEG recordings to the performance timeline.

Additionally, a second level of verification was performed through manual annotation (author A.A.). This involved a posteriori visual inspection of all recordings from the 16‐h performance, including eye‐tracking videos and a top‐down wide‐angle recording of the performance space. This step was critical to determine the time windows during which each participant was physically present in the performance space, as EEG setup occurred in a nearby location where event markers could still be received. This manual verification also proved useful in cases where recording interruptions resulted in multiple EEG recordings per participant. For EEG analyses, we only included datasets with at least 20 min of continuous recording during periods in which the participant was verified to be present in the performance space. In some cases, participants forgot to select some of the individual sections they had attended or selected sections in the post‐performance questionnaire that they had not actually attended. The latter data were excluded from the analyses.

To quantify gaze behavior, we only included individual performances that were attended for their entire duration, using eye‐tracking timestamps to determine attendance percentages for each individual performance. Each eye‐tracking recording provided data on the universal Unix timing of the start and end of the recording and of each event, timings of fixation points, and reception of notifications sent via API. We used Unix timings to determine which participant attended which performance sections. Participants were considered to have attended a performance section if their entering time was before the ending time of the section, and if the time they left the room was after the beginning of the section. As the performance was continuous, participants could arrive partway through sections; to account for this, for each participant–performer pair, we computed a percentage that represents the percentage of the length of the performance section each participant attended. For example, a participant arriving and leaving before an individual performance would have an attendance score of 0%; a participant entering the room 5 min after the start of an individual section lasting 20 min would have an attendance score of 75%; a participant entering the room before the start of a section and leaving after its end would have an attendance score of 100%; and so on. Only participant−performer pairs with an attendance score of 100% were included in the gaze analyses.

### 2.8 Eye‐Tracking Data Preprocessing and Computing Audience Gaze Direction

In total, we collected 83 usable eye‐tracking recordings. Fifty‐one of these participants also completed both questionnaires. After excluding data from six participants due to noisy fixations, the final sample consisted of 45 participants. To analyze the targets and timings of participants’ gaze patterns, we applied the DensePose deep learning model [48]. This model detects human bodies in video frames and returns the coordinates of their location. The complete video recordings were given to the model as inputs. We compared this output with the eye‐tracking data containing the location of the gaze for each frame, thereby detecting the frames where the participants’ gaze coincided with the location of a human body.

For each participant–performer pair, we used the duration of attendance (start and end of the individual performance for the performer and entering and leaving the studio for the participant) to select the frames of the participant video recording that contained only the performer's individual performance. Then, we computed a percentage representing the number of frames in which the participant was looking at people in the scene, relative to the total number of frames for this participant video and each performer witnessed. The resulting measure, referred to as “looking at people in the scene,” captures gaze directed toward any human body detected in the participant's field of view, including both performers and, potentially, the backs of audience members seated in front of the participant. This measure operationalizes the behavioral dimension of attention: the degree to which each audience member directed their visual attention toward others during each individual performance.

### 2.9 EEG Preprocessing

Over the course of the 2‐day performance, 77 members of the audience took part as EEG participants. After a 34% data loss due to technical issues, only 51 EEG participants (25 Saturday, 26 Sunday) were retained with a minimum length of 20 min of continuous recording within the performance space. From those 51 EEG datasets, we followed our established preprocessing protocol from previous work [8] using the EEGLAB toolbox in MATLAB. Mastoid electrodes were excluded prior to analysis. EEG data were downsampled to 250 Hz and high‐pass filtered at 1 Hz. Independent component analysis, as implemented in EEGLAB (default extended InfoMax), was used for visual inspection and artifact removal. Independent components were inspected by a team of researchers (S.A.‐H., A.A., and A.K.) and were marked as artifacts based on a combination of topography, time‐series amplitude values, and power spectrum visualizations in EEGLAB. EEG data and scripts for the EEG preprocessing pipeline, including a list of the components removed through independent component analysis (ICA), are available on OSF (see Data Availability Statement).

After visual inspection and artifact rejection, five more datasets were excluded due to extremely poor signal‐to‐noise ratio. The remaining datasets were low‐pass filtered using a 44 Hz cutoff, trimmed to include only complete sections (i.e., excluding segments in which a participant entered midway through a section; see data index on OSF) and re‐referenced to the average. Data epoching was applied at the individual participant level, segmenting continuous recordings into performer‐specific sections during which the participant was present. Within each performer's section, epochs were further subdivided into music and no‐music sections, reflecting the alternating structure of subsections with and without music while the same performer remained on stage. In total, 46 EEG participants were included in the EEG power analysis, with an average recording time of 68 min and four different performers watched (see Table S5). EEG participants watched on average 18 performance sections (SD = 7, range 4–31), yielding 269 sections in total across the 2‐day performance. Sections were encoded as a combination of performer or group number and subsections with (m) or without (s) music (e.g., p23_m71 or g2_s27). This section‐level data structure formed the basis for all subsequent EEG power analyses.

### 2.10 Correlations Between Gaze, Questionnaires, and EEG

To understand the relationship between felt connection with performers and how engaged the participants were, we calculated Spearman correlations between how much participants looked at others during each individual performance and their ratings of that section, that is, felt social connection, self‐reported focus on the performer, and familiarity with the performer. For the open‐ended questions “Tell us what happened?” and “Tell us how you felt?”, we included word count and sentiment analysis measures (positive/negative valence of each description). Furthermore, we included all pre‐performance measures, that is, trait mind wandering, empathy, Big Five, and both dance experience measures (ODE/PDE). To account for multiple comparisons, we applied Benjamini−Hochberg correction. Additionally, as participants were not attending all individual performances for their entire duration, only observations for wholly attended performances were kept (Attendance = 100%), to be consistent with EEG analysis.

We computed linear mixed‐effects models to account for the nonindependence of repeated ratings within participants. We computed three models, each with “looking at people in the scene” as the predictor and one of the post‐performance ratings as the predicted variable using *lme4* [49] and *lmerTest* [50] packages. All four variables (looking at people in the scene, felt social connection, self‐reported focus, and familiarity) were entered as raw values on their original 0–100 scales; centering was not applied as all variables have interpretable zero points and models included a single predictor each, precluding collinearity concerns. All models included random intercepts for participants to account for the nonindependence of repeated ratings within participants. Random slopes for the gaze predictor could not be estimated reliably. Models either failed to converge or produced singular fits, consistent with the limited number of observations per participant (mean = 3.3 ratings per participant). Random‐intercepts–only models were, therefore, retained for all analyses. Mixed‐effects models assumed normally distributed residuals and independence of random effects across participants. Given the sample size and the use of maximum likelihood estimation, the models are considered robust to moderate departures from normality. No violations of the independence assumption were anticipated given the single‐level clustering structure (ratings nested within participants).

To test whether the effect of looking at people in the scene on felt connection was mediated by self‐reported focus, we conducted a mediation analysis using the *lavaan* package [51], with cluster‐robust standard errors (maximum likelihood estimation with robust standard errors (MLR) estimator, Satorra−Bentler test [52]) to account for the nonindependence of repeated ratings within participants (cluster = participant ID, *N* = 40 clusters, 134 observations). Confidence intervals for the indirect effect were derived analytically from the robust standard errors. All behavioral and questionnaire analyses were performed in R.

We computed EEG power spectra using Welch's method (*pwelch* [53]); for each subsection, that is, each performer split into subsections with and without music, the continuous EEG signal was divided into overlapping 1‐s windows tapered with a Hanning window (50% overlap) [54]. Power spectral density (PSD) was estimated for each channel using a fast Fourier transform length equal to the next power of two of the window size. We then averaged the power values for five frequency bands (delta, 1−4 Hz; theta, 4−8 Hz; alpha, 8−13 Hz; beta, 13−30 Hz; gamma, 30−44 Hz), to compute power for each participant by performer section, subsection (with music/without music), frequency band, and electrode.

For the correlation and mixed‐effects analyses linking EEG to questionnaire ratings, alpha power was then collapsed across channels (median value) and averaged separately for each performer and subsection with and without music, yielding one value per participant−performer pair. This aggregation level was chosen to match the post‐performance ratings, which were collected only once per performer rather than per performance subsection. We computed Spearman correlations between each rating and alpha power. Separate linear mixed‐effects models were computed for each questionnaire rating (felt social connection, subjective focus, familiarity) and for “looking at people in the scene,” with alpha power entered as the sole fixed‐effect predictor in each model, and random intercepts for participants to account for repeated measures. Delta and gamma power were collapsed and averaged in the same way for exploratory analyses; Spearman correlations between each rating and delta/gamma power, and separate linear mixed‐effects models were computed using the same structure as for alpha power. EEG power spectral analyses, including comparisons between subsections with and without music, were performed using MATLAB and the Statistics and Machine Learning Toolbox. EEG‐post‐questionnaire and EEG‐gaze correlations and mixed‐effects models were performed in R.

Unless otherwise specified, correlations reported below in Section 3.1 are computed on the full set of 134 observations from *N* = 40 participants (Figure 3) and treated as descriptive, with Benjamini–Hochberg correction applied for multiple comparisons. Additionally, the EEG correlations use different, smaller samples (*N* = 34 for EEG–questionnaire; *N* = 40 for EEG–gaze). Non‐independence arising from repeated observations within participants is addressed at the confirmatory stage: correlations bearing on the study's primary hypotheses were followed up with linear mixed‐effects models including a random intercept for participant, and, where relevant, cluster‐robust or crossed random‐effects approaches accounting for performer identity.

**FIGURE 3:**
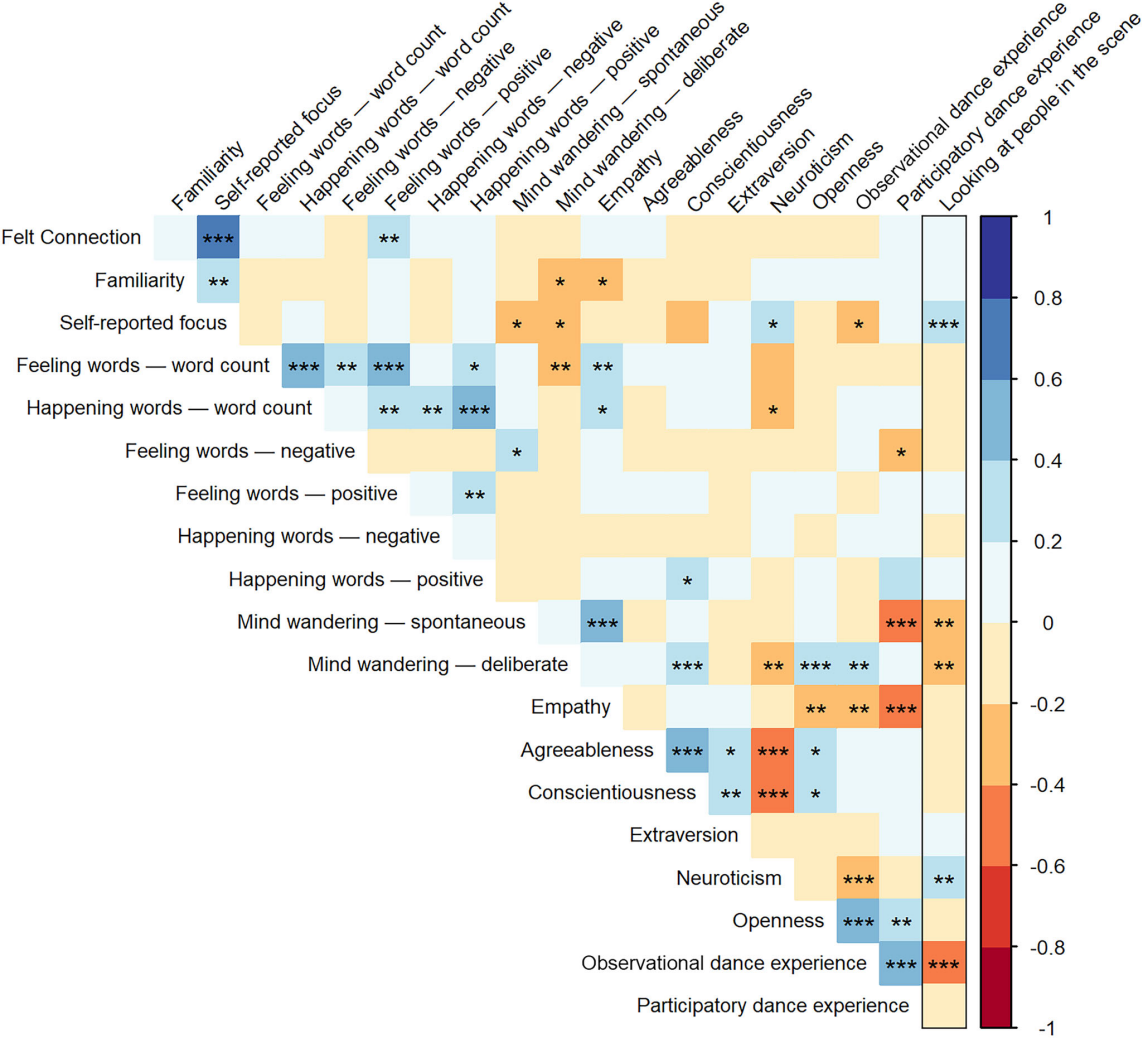
Heatmap of correlations between post‐performance questionnaire ratings, pre‐performance trait measures, and gaze (*N* = 40). Each cell represents the Spearman correlation coefficient between the variable on the row and the variable on the column. Color indicates the direction and strength of the correlation: blue cells indicate positive correlations and orange/red cells indicate negative correlations, with color intensity proportional to the absolute correlation value (scale shown on the right, ranging from −1 to +1). Asterisks indicate significance after Benjamini−Hochberg correction for multiple comparisons: * *p* < 0.05; ** *p* < 0.01; *** *p* < 0.001. Variables include all pre‐/post‐performance ratings and the behavioral gaze measure (“Looking at people in the scene”: percentage of frames in which the participant was looking at other people), highlighted with a black frame.

### 2.11 EEG Power in Subsections With and Without Music

Based on the average PSD per frequency band, we compared EEG power during subsections with and without music using a Wilcoxon signed‐rank test on participant‐level means, across all 46 participants.

Additionally, as an exploratory analysis, we examined the scalp topography of EEG power differences between music and no‐music subsections. To do so, we defined nine brain regions based on electrode positions along two dimensions: frontality (frontal, central, occipital) and laterality (left, midline, right). Each region grouped two electrodes (frontal‐left: F7, F3; frontal‐midline: Fpz, Fz; frontal‐right: F4, F8; central‐left: FC5, C3; central‐midline: FC1, FC2; central‐right: FC6, C4; occipital‐left: P7, O1; occipital‐midline: Pz, POz; occipital‐right: P8, O2). In total, 18 of the 32 recorded electrodes were included in this analysis. First, we averaged EEG power per participant, condition (music, no‐music), and band (alpha, delta, gamma). Second, band power values were log‐transformed to address the positive skew typical of EEG power distributions. Third, log‐transformed values were averaged within each region by combining the two corresponding electrodes. Prior to repeated‐measures ANOVA, region values were screened for outliers using the 1.5 × IQR criterion; outliers were sparse and confined to two participants in occipital regions, and all values were retained in the main analysis. Normality of region distributions was assessed using Jarque–Bera tests; all frontal and central regions of interest (ROIs) met the normality assumption, with minor violations observed in occipital regions attributable to the same two participants. Given the sample size (*N* = 46), repeated‐measures ANOVA was considered robust to these deviations. Sphericity was assessed and found to be violated; Greenhouse–Geisser corrections were, therefore, applied. Finally, a 2 (Music, No‐music) × 3 (Frontality) × 3 (Laterality) repeated‐measures ANOVA was conducted using the *ranova* function in MATLAB.

Beyond the music contrast, we also examined whether the number of performers on stage modulated EEG power. We compared EEG power during individual and group performances of the entire show using a Wilcoxon signed‐rank test on participant‐level means, including only participants who had seen at least one group and one individual performance (*N* = 26).

## 3 Results

We first report associations between gaze and felt social connection (H1), the mediation analysis of this relationship with subjective focus (H2), and the effect of music presence on alpha power (H3). Exploratory analyses included Spearman correlations between all variables and individual difference measures, the effect of music presence on delta and gamma power, and the ANOVA examining scalp topography of music‐related power changes.

### 3.1 Correlation and Regression Analysis

All primary outcome variables were measured on 0–100 scales: felt social connection (mean = 63.72, SD = 27.52), subjective focus (mean = 69.63, SD = 24.73), and prior familiarity (mean = 10.18, SD = 25.25). “Looking at people in the scene” ranged from 33.22% to 87.40% (mean = 61.28%, SD = 13.40%). All continuous predictors were entered on their original scales without centering, as all variables have interpretable zero points and each model included a single predictor, precluding collinearity concerns.

We found that the more people reported positive feelings, the more they wrote about how they felt (*rho* = 0.451, *p* < 0.001) and what happened (*rho* = 0.315, *p =* 0.001), and the more they felt connected with the performer (*rho* = 0.312, *p* = 0.002) (Figure 3). There was a positive correlation between self‐reported focus on the performer and felt social connection with the performer (*rho* = 0.728, *p* < 0.001). There was also a positive correlation between self‐reported focus on the performer and knowing the performer beforehand (familiarity) (*rho* = 0.278, *p* = 0.006), and finally between self‐reported focus and time spent looking at people in the scene (*rho* = 0.355, *p* < 0.001) (see Figure 3). Our findings, therefore, reveal a link between attention, engagement, and felt social connection. While we did not find a significant correlation between felt connection and looking at people in the scene, a linear mixed‐effects model with random intercepts for participants revealed a borderline association between these two variables (*B* = 0.37, *SE* = 0.20, *t*(84) = 1.85, *p* = 0.068), falling just short of conventional significance thresholds.

To further investigate the effect of “looking at people in the scene” on felt connection, we conducted a mediation analysis, with self‐reported focus as a mediator on the effect of “looking at people in the scene” on connection felt with the performer. “Looking at people in the scene” significantly predicted focus ratings (*B* = 0.786, *R* ^2^ = 0.181, *p =* 0.002). Self‐reported focus also remained a strong predictor of felt connection (*B* = 0.895, *p* < 0.001). The indirect effect of “looking at people in the scene” on felt connection via self‐reported focus was significant (indirect = 0.70, 95% CI [0.20, 1.21], *p* = 0.006). The direct effect of gaze on felt connection was nonsignificant after accounting for focus (*B* = −0.278, *p* = 0.13) (see Figure 4). These results show that the relationship between “looking at people in the scene” and felt social connection was mediated by self‐reported focus on the performer.

**FIGURE 4:**
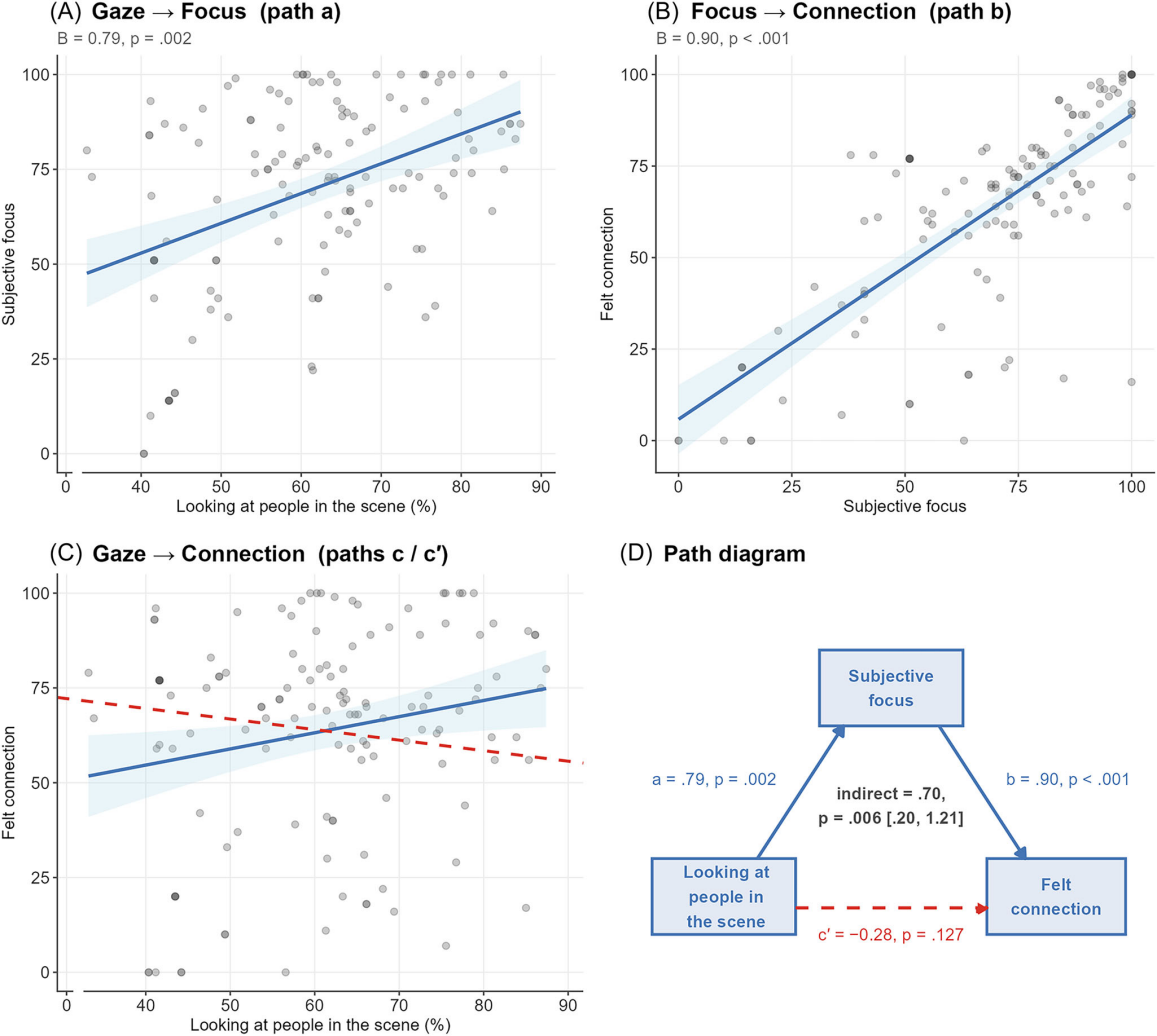
Mediation of the relationship between looking at other people in the scene and felt social connection by subjective focus. Panel A shows the relationship between looking at people in the scene (%) and subjective focus (path a). Panel B shows the relationship between subjective focus and felt social connection (path b). Panel C shows the total and direct relationship between looking at people in the scene and felt social connection, before and after accounting for the mediator. Panel D shows the estimated mediation model. Each point in panels A–C represents one participant–performer observation. Regression lines are shown with 95% confidence intervals. All paths are estimated using cluster‐robust standard errors (MLR estimator) to account for the nonindependence of repeated ratings within participants (*N* = 40 participants, 134 observations). Regression coefficients (*B*) and *p*‐values are reported. The indirect effect (*a* × *b* = 0.70, 95% CI [0.20, 1.21], *p* = 0.006) was significant, whereas the direct effect (*c*′ = −0.28, *p* = 0.127) was not. This pattern supports an indirect association through subjective focus but does not establish complete or causal mediation.

Prior familiarity correlated positively with self‐reported focus but showed no significant association with felt social connection or any engagement variables (self‐reported positive feelings, self‐reported negative feelings and word count). In fact, most of the participants did not know any of the performers beforehand (see Figure 5).

**FIGURE 5:**
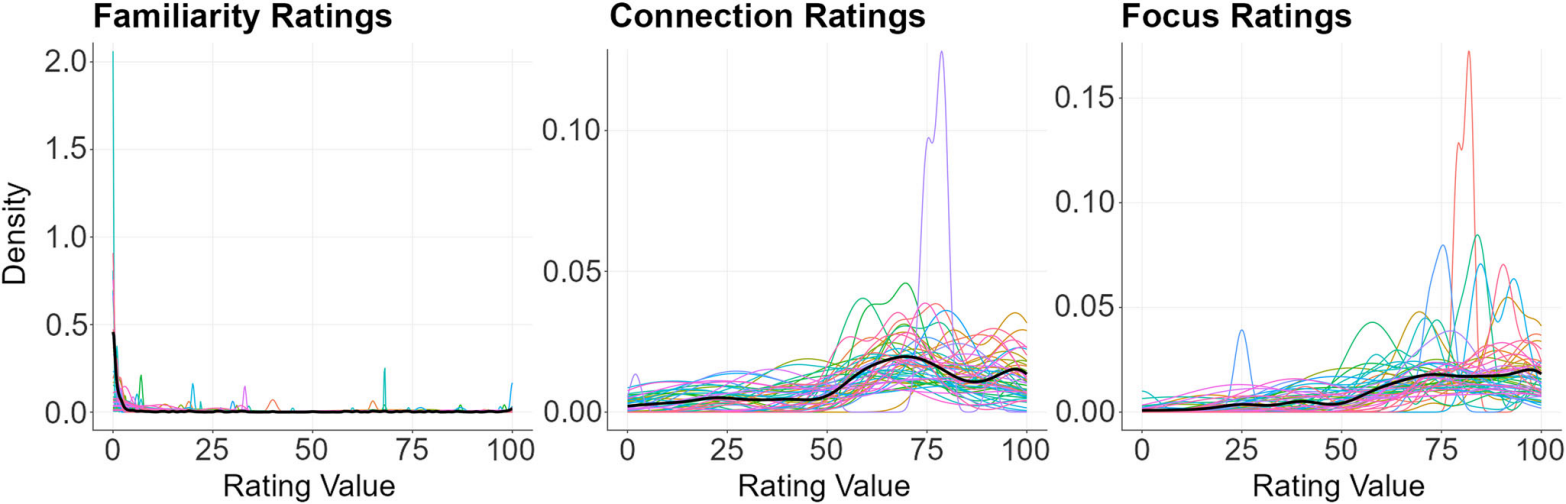
Distribution of post‐performance ratings of prior familiarity, felt social connection, and subjective focus across all 50 performers. Panels show the distribution of ratings for prior familiarity with the performer (left), felt social connection with the performers (center), and subjective focus on the performer (right). The *x*‐axis represents the rating value on a 0–100 scale, from “Not at all” (0) to “Very much” (100). The *y*‐axis represents density. Each colored line corresponds to the distribution of ratings given by all audience members who attended one performer's section (*N* = 50 performers). The thick black line depicts the mean density across all performers.

Spearman correlations between EEG power and ratings (*N* = 34) revealed significant associations between alpha power and both felt social connection (*rho* = −0.109, *p* = 0.031) and subjective focus (*rho* = −0.129, *p* = 0.013), surviving the Benjamini−Hochberg correction. To account for the fact that each participant contributed multiple ratings across performers, we followed up each significant correlation with a linear mixed‐effects model with random intercepts for participants, with each rating as the outcome and EEG power as the sole fixed‐effect predictor. However, none of the significant Spearman correlations were confirmed in linear mixed‐effects models predicting ratings from alpha power. Exploratory analysis revealed significant correlations between delta power and subjective focus (*rho* = −0.122, *p* = 0.017) and familiarity (*rho* = −0.154, *p* = 0.003), surviving the Benjamini−Hochberg correction. Similarly, gaze toward performers correlated positively with alpha power (*rho* = 0.209, *p* < 0.001) and delta power (*rho* = 0.116, *p* = 0.004, *N* = 40), both surviving the Benjamini−Hochberg correction, but neither association survived in mixed‐effects models.

The gaze behavior of spectators is also impacted by individual differences. Audience members prone to mind wandering, whether spontaneous (*rho* = −0.301, *p* = 0.002) or deliberate (*rho* = −0.295, *p* = 0.003), looked at people in the scene less frequently. Conversely, those who score higher on neuroticism looked more often at people in the scene (*rho* = 0.295, *p* = 0.003). Unexpectedly, we also found that spectators with greater ODE looked less at people in the scene (*rho* = −0.452, *p* < 0.001).

We observed a few significant correlations between individual differences and ratings from the post‐performance questionnaire. While felt social connection ratings did not depend on individual differences measures, focus ratings were negatively correlated with spontaneous (*rho* = −0.256, *p* = 0.012) and deliberate (*rho* = −0.26, *p* = 0.011) mind wandering and ODE (*rho* = −0.235, *p* = 0.024). Interestingly, focus ratings were positively correlated with neuroticism (*rho* = 0.242, *p* = 0.02). Additionally, the more participants are prone to deliberately mind wander, the less words they wrote about how they felt (*rho* = −0.266, *p* = 0.009). Empathy was also positively correlated with how much spectators wrote about what happened (*rho* = 0.247, *p* = 0.016) and how they felt (*rho* = 0.318, *p* = 0.001) (see Figure 3). To see all Spearman correlation results, see Tables S6 and S7.

### 3.2 EEG Power in Subsections With and Without Music

We compared EEG power between subsections with and without music, as well as moments where a group was performing compared to when only one performer was on stage. Alpha power was significantly lower during music sections (*Z* = −3.518, *p* = 0.0004), consistent with H3. Exploratory analyses revealed that delta power was significantly higher during music sections (*Z* = 2.983, *p* = 0.0029) and gamma power was significantly higher during music sections (*Z* = 3.431, *p* = 0.0006) (see Figure 6).

**FIGURE 6:**
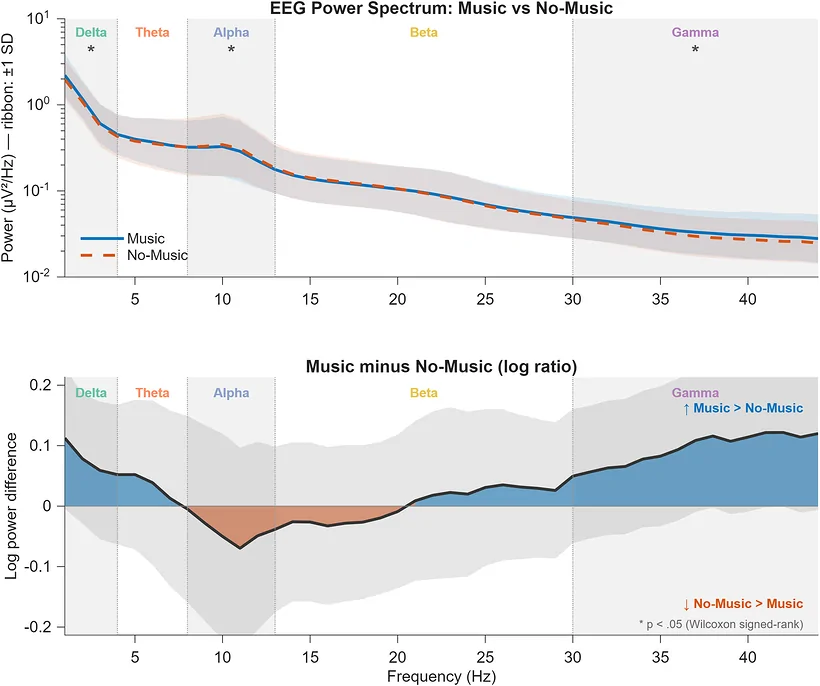
Grand‐average EEG power spectra and log power differences between music and no‐music sections. Grand‐average EEG power spectra and difference plot across 46 participants. Top panel: Geometric mean power spectral density (µV^2^/Hz) plotted on a logarithmic scale for music (solid blue line) and no‐music (dashed orange line) conditions across the full recorded frequency range (1–44 Hz). Shaded ribbons represent ±1 SD across participants. Vertical dotted lines demarcate canonical frequency bands (delta, theta, alpha, beta, gamma). Gray shading indicates frequency bands showing significant condition differences (Wilcoxon signed‐rank test, *p* < 0.05): delta (1–4 Hz), alpha (8–13 Hz), and gamma (30–44 Hz). “*” indicates bands with significant power differences between conditions. Bottom panel: Log power ratio (Music minus No‐Music) computed as the difference between log‐transformed geometric means. Positive values (blue fill) indicate higher power during music; negative values (orange fill) indicate higher power during no‐music. The gray ribbon represents ±1 SEM around the difference. Gray shading, vertical lines, and band labels follow the same conventions as the top panel.

To assess the topography of these effects, we conducted a repeated‐measures ANOVA with factors Condition (Music, No‐music), Frontality (Frontal, Central, Occipital), and Laterality (Left, Midline, Right) on log‐transformed power. For the delta band, significant main effects of Condition (*F*(1,45) = 12.59, *p* = 0.001), Frontality (*F*(2,90) = 85.69, *pGG* < 0.001), and Laterality (*F*(2,90) = 42.68, *pGG* < 0.001) were observed, as well as a significant Frontality × Laterality interaction (*F*(4,180) = 23.52, *pGG* < 0.001), indicating strongest delta power over occipital and midline electrodes. No interactions involving Condition were significant (all *pGG* > 0.06). A similar pattern was observed for the alpha band, with significant main effects of Condition (*F*(1,45) = 4.90, *p* = 0.032), Frontality (*F*(2,90) = 62.40, *pGG* < 0.001), and Laterality (*F*(2,90) = 33.33, *pGG* < 0.001), and a significant Frontality × Laterality interaction (*F*(4,180) = 15.33, *pGG* < 0.001), again indicating strongest power over occipital and midline electrodes. No interactions involving Condition were significant (all *pGG* > 0.37). The gamma band exhibited similar results, with a significant main effect of Condition (*F*(1,45) = 9.98, *p* = 0.003), confirming higher gamma power during music sections. Significant main effects of Frontality (*F*(2,90) = 28.69, *pGG* < 0.001) and Laterality (*F*(2,90) = 5.48, *pGG* = 0.008) were observed, as well as a Frontality × Laterality interaction (*F*(4,180) = 15.38, *pGG* < 0.001). No interactions involving Condition were significant (all *pGG* > 0.30). Topographical maps (Figure 7) showed a pronounced posterior distribution of activity across all three frequency bands, with higher power over occipital regions relative to frontal sites. This spatial pattern was highly similar between music and no‐music conditions.

**FIGURE 7:**
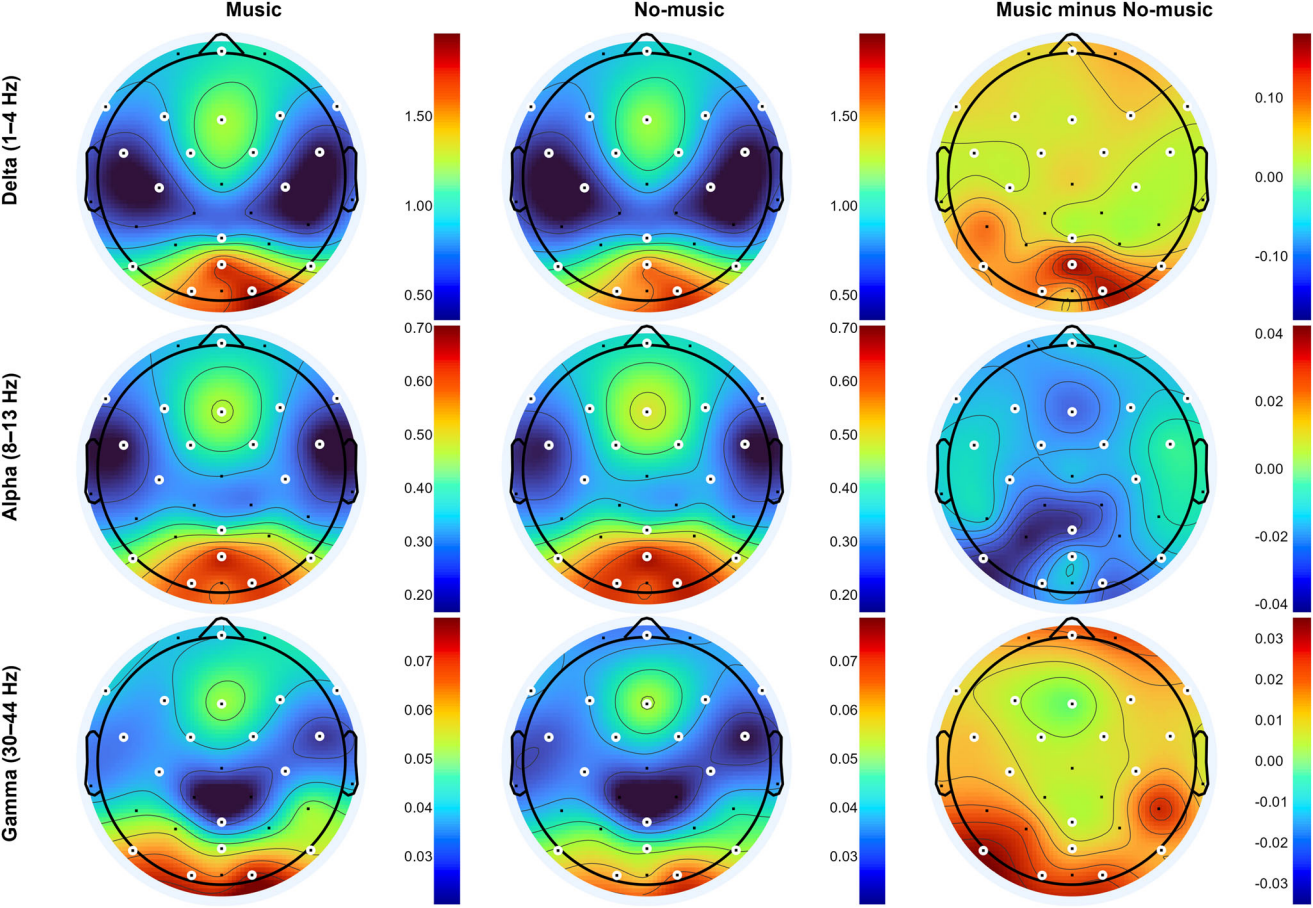
Scalp topography of EEG power during music and no‐music conditions. Topographical maps of EEG power (µV^2^) averaged across 46 participants, separately for the delta (1–4 Hz; top row), alpha (8–13 Hz; middle row), and gamma (30−44 Hz; bottom row). Each row contains three panels. The left panel shows grand‐averaged power during the music condition; the center panel shows grand‐averaged power during the no‐music condition; both are plotted on a shared color scale where warm colors (red) indicate higher power and cool colors (blue/purple) indicate lower power. The right panel shows the difference map (music minus no‐music), plotted on a symmetric color scale centered at zero, where red indicates higher power during music and blue indicates higher power during no‐music subsections. Maps are interpolated across all 32 recorded electrodes and displayed in standard head view (nose pointing up, left ear to the left). All 32 electrodes are displayed as black dots; the 18 electrodes included in the statistical analysis are marked with a white circle. For the gamma band, electrode CP5 was replaced by the mean of neighboring electrodes (FC5, C3, P7, CP6) due to atypically elevated variance (0.09 µV^2^) relative to all other electrodes.

Exploratory analyses comparing group and individual performances revealed no significant difference for alpha or delta power, but theta power was significantly higher during group sections (Wilcoxon signed‐rank test, *Z* = 2.832, *p* = 0.005).

## 4 Discussion

In this large‐scale real‐world live performance study, we set out to study if engagement with live dance depends on experiencing social connection with dance performers. Prior research suggests that dancing and making music together is associated with group bonding between participants and signals affiliation to others [2, 3, 55, 56], but the role that social affiliation plays for audience engagement is unknown.

### 4.1 Social Connection, Gaze, and Engagement

In this study, we showed that higher felt social connection is associated with reporting more positive feelings after the show, and participants who reported more positive feelings also wrote more about how they felt and about what happened during the performance. Indirectly, felt social connection was measurable as increased looking at people in the scene, mediated by self‐reported focus on individual performers. Our findings align with prior research on social influences on gaze: people look more at people they like [21, 24, 25], and extend these findings to the performing arts, specifically live dance. It is important to note that both subjective focus and felt social connection were measured retrospectively, after the performance. The mediation, therefore, described a covariance structure in post‐hoc reflective reports rather than a moment‐to‐moment process unfolding during the performance itself. These results suggest that engagement with the dance performance may not be fully disentangled from engagement with the dancer themself.

Engagement with live performance also depends on expertise [7] and on kinship between performers and spectators [57]. In this study, we showed that prior familiarity is not a prerequisite to develop a felt social connection [7, 14]. This finding is consistent with the artistic intentions underlying *How Shall We Begin Again?*, which explicitly foregrounded social connection and mutual recognition between performers and audience members; what Fong describes as “making the spaces between people available” to connection. Most of our spectators did not know any of the performers, suggesting that felt social connection with a performer arose during the performance itself, perhaps as a consequence of their individual investigation into the live moment shared with the audience. Our findings suggest that the potential of live dance to generate feelings of social connection is measurable as distinct gaze behavior of audience members.

Interestingly, spectators prone to mind wandering looked at others less, reported focusing less on the performer, and provided less detailed accounts of the feelings they experienced during the show. A tendency to mind wander was associated with reduced gaze toward people and lower self‐reported focus, which aligns with previous research indicating that mind wandering is associated with decreased attention to external stimuli and social cues [58]. Surprisingly, participants with more observational dance experience tended to look less at and reported focusing less on performers, although this did not impact their overall ratings of the performance. While speculative and not directly tested in the current study, this pattern might reflect a sparser engagement style, where prior expertise allows experienced spectators to process information more efficiently and with greater focus [58]. Perhaps experienced spectators were able to distribute attention more broadly across contextual aspects of the performance [60, 61], whereas less experienced spectators focused primarily on the individual performer. Relatedly, experienced spectators may engage in a more active, reflective mode of reception: simultaneously watching and interpreting the performance, rather than passively absorbing it. Finally, the positive association between neuroticism and both gaze and self‐reported focus may reflect the heightened sensitivity to social cues characteristic of neurotic individuals [62]. Greater trait empathy was associated with writing more about individual performers, further supporting the idea that engagement is linked to felt connection with a performer and aligns with past research suggesting that personality traits predict gaze behavior when looking at faces [63]. Together, our results showed that audience engagement is shaped by a complex interplay of a spectator's personality and prior experience with what happens on stage and who is performing.

### 4.2 EEG Arousal and Social Connection

EEG results revealed modest but significant relationships between alpha power, and both felt social connection and subjective focus, as well as between delta power and subjective focus and familiarity, all surviving correction for multiple comparisons. However, none of these associations survived in linear mixed‐effects models, suggesting these relationships are driven primarily by between‐participant rather than within‐participant differences. This pattern is consistent with the known sensitivity of alpha power to individual differences in arousal and attentional style [64], rather than reflecting moment‐to‐moment fluctuations in social engagement with individual performers. However, the small sample size of the EEG‐questionnaire subset and low statistical power limit any firm conclusions.

EEG alpha power did depend on the presence of music during individual performances. Alpha power was higher when there was no music, consistent with reduced arousal and attentional demand in the absence of sound [12, 13]. Moreover, delta power was higher during music sections, consistent with the role of delta oscillations in auditory and rhythmic processing [65, 66]. Gamma power was equally higher during music sections, consistent with increased auditory cortical processing during music listening [67], anticipatory beat processing [68], and top‐down attentional engagement in response to musical stimulation [29, 69]. However, topographic analyses revealed a largely occipital focus of alpha, delta, and gamma power, suggesting that the effect of music was more indirect and supported sustained visual engagement with the performance, consistent with the modulation of posterior alpha by visual attention [13]. Future work using video recordings of the performances and in more controlled, higher‐density EEG recordings could apply source localization to better understand the origin of these findings and their relationship to specific musical features.

Finally, theta power was significantly higher during group sections compared to individual performances, suggesting that the number of performers on stage may modulate neural activity in ways not captured by alpha or delta power alone. Theta has been linked to social processing [70] and social exclusion [71, 72]; future work could explore whether theta band activity is modulated by the number of people looked at. It is worth noting, however, that group sections in this performance were characterized not by synchronized ensemble movement but by multiple performers simultaneously engaging in distinct actions, confronting audience members with a complex, multiagent scene requiring continuous attentional choices about where to direct their gaze [73, 74]. Elevated theta during these sections may, therefore, reflect not only increased social processing demands but also the cognitive load associated with navigating a visually complex and unpredictable scene, consistent with theta's broader role in effortful cognitive processing [75]. Disentangling these contributions (social, attentional, and perceptual) would require designs that independently manipulate group size, movement synchrony, and scene complexity.

### 4.3 Limitations

In this real‐world neuroscience study, we attempted to capture social influences on engagement with live dance. The durational nature of *How Shall We Begin Again?* as a series of nonrepeatable, individual improvised performances served to maximize physical and social liveness of the event, yet posed several technical challenges to data collection, synchronization, and analysis.

First, our measure of gaze behavior does not distinguish between looking at performers and looking at other audience members. This is a common issue with automatic analyses of attention targets [76, 77]. Nonetheless, in a control analysis incorporating seating position, we found no significant difference in the amount of time spent looking at others between participants seated in the first row and participants seated in rows 2, 3, or 4 (*rho* = 0.15). Moreover, any influence of looking at other spectators rather than performers would likely have reduced the relationship between self‐reported focus and looking behavior rather than increased it. Therefore, we argue that our measure of “looking at people in the scene” is a reliable proxy for looking at performers.

Second, not to interrupt the audience's continuous experience of the show, we assessed engagement only retrospectively and relied on the spectators’ memory of the event rather than their experience in the moment, which can produce systematic bias [14]. Audience ratings reflect summative evaluations of the entire show, rather than moment‐to‐moment engagement with the unfolding performance. Importantly, we identified and corrected memory errors using manual annotations of the eye movement sensors. These errors were scarce and, therefore, not systematically analyzed, but it seems possible that felt social connection with a performer would impact memory for a performer. Despite the temporal and physical distance between our self‐report measures and the actual show, we were in fact able to establish clear associations between continuous measures of audience engagement (looking behavior) and memory‐based reports of social connection and engagement, highlighting the potentially long‐lasting impact attending this live show can have on audience members.

Third, to keep the post‐performance questionnaire as concise as possible, we used single‐item measures of focus, connection, and prior familiarity rather than multiple‐item questionnaires as measures of aesthetic engagement (i.e., aesthemos [78]). Additionally, to match the numerical scales and to avoid confusion between connection to audience members and performers, we did not use alternative, single‐item measures of social connection, such as the other‐in‐self scale [79]. Nonetheless, we observed meaningful relationships between our single‐item measures and validated psychometric assessments of mind wandering, empathy, and dance experience, suggesting that our single‐item measures can meaningfully tap into attentional focus, social connection, and prior familiarity with performers.

Additionally, we did not record data from dance performers in this study and do not know to what extent the performers experienced a social connection with the audience. Our EEG analyses are limited by relatively small sample sizes and insufficient accuracy of synchronization between eye movement sensors and EEG devices to allow for time‐resolved analyses of continuous audience experience. As a result, our comparison of subsections with and without music did not account for musical features over time and did not allow us to compute measures of interpersonal brain synchrony. Future, more controlled laboratory work involving video recordings of *How Shall We Begin Again?* should allow us to link brain activity to music and movement features and allow for a more fine‐grained analysis of underlying brain sources.

Finally, wearing eye‐tracking glasses or mobile EEG may have influenced gaze behavior, as awareness of being eye‐tracked has been shown to induce social‐norm‐based modifications to natural‐looking behavior; specifically, participants who know their eyes are being tracked tend to look less at others’ bodies compared to those who are unaware [80]. Similarly, wearing physiological equipment may have introduced some self‐consciousness. To minimize disruption to the audience experience, the experimental aspect of the performance was discussed at length with the choreographer and performers prior to data collection, and several practical measures were taken: participants were carefully accompanied from their entry to departure of the building, and eye‐trackers were referred to as “eye movement sensors” to reduce the feeling of being watched. In a previous study, we showed that participants wearing mobile EEG were indeed less engaged when watching dance, but that this effect disappeared when prior dance experience was taken into account [7]. The broadly positive self‐reported engagement and connection ratings observed here are consistent with this view, suggesting that the ecological validity of the live performance context was largely preserved despite the instrumentation. Future work could more directly examine this question by comparing instrumented and noninstrumented audience members attending equivalent performances.

## 5 Conclusion

Together, our findings show that the experience of a live performance is shaped by a dynamic interplay between subjective felt social connection, attention, and individual differences between audience members. By combining mobile EEG, eye‐tracking, and self‐report in an ecologically valid performance setting, this study demonstrated the feasibility of investigating social cognition in naturalistic settings. Our results highlight that the experience of live dance depends not only on what is being performed (dance/choreography) but also on who is performing. As real‐world cognitive neuroscience expands beyond pairs of people, our research allows us to investigate questions that are difficult if not impossible to pose in laboratory settings. Studying the complex social mechanisms that are at play when people attend and participate in dance provided a rich, yet replicable research context to understand the behavioral and physiological processes that underpin human social connection in the real‐world.

## Author Contributions

Conceptualization: A.A., L.A.R., H.L., M.S., J.A.W., D.C.R., and G.O. Data curation: A.A., A.K., S.A.‐H., M.F., L.A.R., and H.L. Formal analysis: A.A., S.A.‐H., M.F., and D.C.R. Funding acquisition: M.S., J.A.W., D.C.R., and G.O. Investigation: A.A., S.A.‐H., M.F., L.A.R., H.L., M.S., J.A.W., D.C.R., and G.O. Methodology: A.A., S.A.‐H., L.A.R., H.L., D.C.R., J.A.W., and G.O. Project administration: M.S., J.A.W., D.C.R., and G.O. Resources: A.A., S.A.‐H., M.F., L.A.R., H.L., M.S., J.A.W., D.C.R., and G.O. Software: A.A., S.A.‐H., M.F., L.A.R., and D.C.R. Visualization: A.A. and M.F. Writing – original draft: A.A., A.K., S.A.‐H., M.S., and G.O. Writing – review and editing: All authors.

## Conflicts of Interest

Several authors (H.L., J.A.W., M.S., and G.O.) also participated as performers in *How Shall We Begin Again?*. While the primary data were collected from audience members and this dual role does not compromise data integrity, we acknowledge the potential for interpretive bias. Formal data analysis was conducted by A.A., S.A.‐H., M.F., and D.C.R., who did not perform in the show, providing an additional safeguard against bias.

## Supporting information




**Supplementary Material**: nyas70412‐sup‐0001‐TableS1‐S7.docx

## Data Availability

All data are available in a BIDS‐formatted dataset on OpenNeuro: doi:10.18112/openneuro.ds007802.v1.0.0. Additional materials, including the EEG preprocessing script, ICA components removed, and the Data Index file, are available on OSF: https://osf.io/fut3r/overview?view_only=58ef7f4c091943f38c81049067f40ada.

## Footnotes

1 Three of the 50 performers chose to perform together with their infant child, transforming their individual live investigations into parent−child duos.
