# Supplementary material for: Audience Gaze and Felt Social Connection With Individual Performers During Live Dance
Source: Ann N Y Acad Sci. 2026 Oct 19;1564(1):e70412. doi: 10.1111/nyas.70412 (PMC13678572; doi:10.1111/nyas.70412)
Supplement: Supplementary file 1 [file NYAS-1564-0-s001.docx]

**Table S1**

*Self-descriptions of the 50 performers, and titles of their solo performances*

| **Title** | **Self-description** |
| --- | --- |
| Stop apologising | Queer / White / Pink eyebrows |
| This is my body. ___ ___ ____. | Gender-confused / Bashkir / Long dark brown hair,cheekbones, blushes, curvy |
| Orbiting Myself as I Orbit Myself | Woman / of Indian heritage / Dark brown hair shaved on both sides and cropped at the top |
| Taking a reading, beginning again | Male / White / Dark blond and grey short hair and beard |
| It's been an endless bad break up & he might be here | Girl / Dad from England, Mum from Germany / Long brown hair with a short fringe, 1m 10cm tal |
| Be body | Male, dark, stubble |
| Climate reparations now. | Queer Woman / White / Brown bob |
| This is me: when I dance it feels like love | Gender non-conforming / Seated dancer / Short hair |
| There's nothing to see here. Nothing is safe. Everything will be fine. | I am a male identified person, with long hair and beard. Tall and thin with a tattoo on my right arm. Latinx and tired of moving around. |
| A version of now | Female / East Asian / High pony tail |
| Catching breath | Man / Mediterranean-looking, light olive skin / Short dark-brown hair with moustache and goatee |
| To share something from myself | Male / Latin Mexican / Sharing a part of me |
| When will you be home again? | Male / South Asian / Beard |
| Maybe there's no need to try so hard, to be here. Ella, Dwi ddim angen trio mor galed, i fod yma. | I am a white Male, I am Welsh. I have brown hair and a stubble. IÕm not tall, really. I have a couple of tattoos one on ( left ) inside my forearm in the shape of a small heart. |
| Ngo zhong yi nei (I like you) | Not Male / East Asian Chinese / Black shoulder length hair; Girl / I speak Cantonese and English with Mummy and Spanish with Daddy/ I have long straight hair |
| I've got a lot to catch up on | Male / White other / Muscly |
| To hold lightly | Woman / Mixed-race Chinese Jamaican heritage / Long dark hair |
| My journey as a dancer | Man / White English / Creative dancer |
| I am realising things | Female / Mixed Afro-Caribbean and White British / Curly hair |
| Here | Xi-mali |
| !~~~***~~~! | Non-binary / Swedish-born Chinese / red and black hair, EEG headset |
| Three-Quarter moon. 'a desire to erase everything extraneous' Olivia Laing. | Woman / Taiwanese-British / Longish greying black hair |
| I am just a Mirage | Male / Person of the world / Has a moustache |
| I dream of being a courageous, soft skinned starfish | Female / White other / Blond shoulder-length hair with a fringe |
| Breathe til I evaporate | They call me a woman... I come from the lands of tropical Savanna in southern Africa. My hair is dark and coily. |
| I want more of this | Female / East Asian / Shoulder length dark hair, medium height |
| The Vibe Machine | Free Gender / Mixed Race, Black African, Black British / I hold a great presence and focus and I energise a lot of people when I'm dancing |
| HOMESICKNESS | Female / Black British / Mid-length sister locks, tall, of a slim build |
| Scribble Rhythm | Kuan-Yu and Kayjay ID |
| Last night at the Jerusalem tavern | Male / White, Scots Irish / Brown hair, slim |
| HELLO HELLO HELLO HELLO HELLO HELLO HELLO | Female / East Asian / Dark brown hair with highlights |
| arriving backwards, slowly tracing the doorway with my grazed hands | Male / White / With a bleach-blonde mop of unruly hair |
| How High the Moon | Woman / Japanese / I am rather small, 153cm |
| Enough With The Interruptions, Softly Textured Edges Await | Woman / White Irish / Long blonde hair that often has lots of pink in it, sometimes other colours |
| If Today Is Tomorrow | Woman / Chinese / Black hair that is kind of ear-length |
| Water | Woman / White with Jewish heritage / Long dark hair with blonde stripe |
| Pause a Circle? | East and Southeast Asian Mixed Race, of Hong Kong heritage/ Short black hair with the back shaved, facial hair on upper lip / Long limbs, lean body type |
| Dawn without Tara | Woman / My skin is sort of pink-white-red and gets really red when I run about / Shoulder-length red-brown auburn-ish hair |
| Can I do it again? Without the fear | Male / White English / My hair is turning grey |
| The potential that therefore I am (goes on) | Woman / Chinese, born and grew up in Australia / Long black hair |
| What if I did not give a fuck about performing and be selfish? | Female / Originally from Germany / Strawberry-blonde hair that is fairly long |
| There | Male / Chinese person from Hong Kong / Black hair, muscular build |
| I'm looking for a sublet | She/her / White Latin American person / Quite tall, skinny, with messy hair which is kind of long |
| If in doubt let it out | Woman / White British / Quite short, with short white hair |
| Humming Cabbage | Man / Chinese / Medium build with short black hair |
| It's for me (It's not for you, but I'm glad you're here). | Non-binary person / White skin / Short brown floppy hair |
| Patient | Woman / White skin / Very short hair |
| You are safe | Female / British Chinese / My hair is straight and dark |
| Just keep swimming | Male / East and Southeast Asian mixed heritage, born in Hong Kong / Dark brown short hair, average height, slim build |
| [in the] currents | Woman / Black British / Mid-height, with afro hair |

**Table S2**

*All songs titles and artists played during individual performances*

| **Song Title** | **Artist** |
| --- | --- |
| CUFF IT | Beyoncé |
| Careless Whisper | George Michael |
| Don't Let Go (Love) | En Vogue |
| Choci Loni | Young Marble Giants |
| Gesi Bağları | Selda Bağcan |
| Oh Yeah | Yello |
| Te Queria | Lido Pimienta |
| Traces Of You | Anoushka Shankar |
| Forces... Darling | Koop |
| The Beginning | RuPaul |
| Heartbeats | The Knife |
| Monument | Röyksopp, Robyn |
| Verbal Fight | shukar collective |
| Stampede | Mikael Karlsson |
| Eden | Hania Rani |
| Bizarre Love Triangle - Extended dance mix | New Order |
| Flame Lily | MA.MOYO |
| Cosmos in Blue | Viken Arman |
| Lou | Rone |
| Spellbound | Siouxie and the Banshees |
| I'm Grateful | Céline Gillain |
| The Now | Anna Wise |
| Movement | Hozier |
| My Witness | The Irrepressibles |
| Body Electric | Lana Del Rey |
| Who We Want To Be | Tom Day |
| Sweet Harmony | The Beloved |
| Nobody's Diary | Yazoo |
| Holm Sound | Erland Cooper |
| Te Queria | Lido Pimienta |
| Hiç | Kiana |
| When We Move | Common, Black Thought, Seun Kuti |
| Free Spirit - Duo Version | Ibrahim Maalouf |
| Still Ray | Raphael Saadiq |
| Amerika | Rammstein |
| Entre dos Aguas | Paco de Lucía |
| Praan | UN Chamber Music Society (Brenda Vongova, Artistic Director) |
| Tick of the Clock | Chromatics |
| Redbone | Childish Gambino |
| Cherry Wine - Live | Hozier |
| Afeto | Mayra Andrade |
| Wild Is The Wind | David Bowie |
| Ysbryd Y Nos | Edward H. Dafis |
| The Man Don't Give A Fuck | Super Furry Animals |
| As It Was | Harry Styles |
| Prickly Pear | Portico Quartet |
| Hai Apna Dil To Aawara - Happy Version | Hemant Kumar |
| Body & Soul | Aki Takase, David Murray |
| This Woman's Work - Live from MTV Unplugged Brooklyn, NY - May 1997 | Maxwell |
| Basshund – Guido edit | Kolsh |
| Last Words of a Shooting Star | Mitski |
| Bye Bye Macadam | Rone |
| Let Me Roll It | Paul McCartney |
| Bitch I'm Madonna | Madonna |
| Sand (feat. Karen Whipple) | Nathan Lanier |
| Dance Monkey | Tones and I |
| Señorita | Shawn Mendes |
| Sexual Healing | Marvin Gaye |
| House Music Is Controllable Desire You Can Own | DJ Sprinkles |
| I Only Said | my bloody valentine |
| Stranger Than Fiction | Moses Boyd |
| Come Rain or Come Shine - Remastered | Art Blakey & The Jazz Messengers |
| Clarach | Seckou Keita |
| How Deep Is Your Love | PJ Morton |
| When Trees Start to Shake | Ryong |
| ライディーン | Yellow Magic Orchestra |
| Lido | Bicep |
| Form By Firelight | Jon Hopkins |
| U Got The Look | Prince |
| Parallel Jalebi | Four Tet |
| Welcome | Harmonia |
| Mirage | Santana |
| Pure Jam | Yellow Magic Orchestra |
| Cleopatra | Nova Twins |
| Bird Gerhl - Live | Antony and the Johnsons |
| Slow Train Coming / License to Kill | Michael Shaeffer |
| Allegory | Throwing Snow |
| Classon | Grenier |
| Melum | Throwing Snow |
| Riot Rhythm | Sleigh Bells |
| 喂呀喂呀 | GTB |
| Seventeen | Sharon Van Etten |
| Sing It Back - Afro Mix | DJ Hermes, Fly |
| Madonna | Hugel, Lovra |
| The Swan | Nwando Ebizie |
| Dem Boy Paigon | J Hus |
| The Thrill Is Gone | B.B. King |
| Bamthathile | Sun-El Musician |
| Daily, Nightly, and Ever So Rightly | Khaya |
| 早安，晨之美！ | Crowd Lu |
| So Proud | Khaya |
| How to be invisible | Kate Bush |
| Bullet With Butterfly Wings - remastered 2012 | The Smashing Pumpkins |
| Kiss | Prince |
| Bizarre Love Triangle - Extended dance mix | New Order |
| Cristobal | Airhead |
| Saturn Song | Beach House |
| Feel It Out | Yaeji |
| Sangre y Mateo | Murcof |
| A Somewhere Place | Vincent Gallo |
| Pinky's Dream | David Lynch |
| 茎 - 日本語バージョン | Sheena Ringo |
| Pygmy Lullaby | Jan Garbarek |
| Suite Bergamasque, L. 75: III. Clair de lune | Claude Debussy |
| Touch | Holy Other |
| Goodbye Horses | Q Lazzarus |
| AIR | SKY H1 |
| 海浪 | deca joins |
| 心要野 | Queen Sea Big Shark |
| 親愛的無情孫小美 | EggPlantEgg |
| Smile | Wolf Alice |
| I Dance Like This | David Byrne |
| Giant | The The |
| Tick of the Clock | Chromatics |
| 荊棘海 | Vincy Chan |
| On My Way | Hins Cheung |
| 弱水三千 | Juno Mak |
| Bette Davis Eyes | Kim Carnes |
| Dark Moon, High Tide | Afro Celt Sound System |
| Lay, Lady, Lay | Bob Dylan |
| Orion | Rodrigo y Gabriela |
| You Are Not Alone | Mavis Staples |
| The Peace Of The Wild Things/Dayblind | Crooked Still |
| Triple Sun | Coil |
| Space, Love | Leila |
| Moody Relish | Maxine Funke |
| Un homme | Jérémy Frerot |
| Let It Out | 福原美穂 |
| Danser encore | HK |
| Pray You Catch Me | Beyoncé |
| Airam | Nil Ciuró |
| East Atlanta Love Letter (feat. Future) | 6LACK |
| Bitch I'm Madonna | Madonna |
| I Feel for You | Chaka Khan |
| City of Light | Fennesz |
| Abolition (Infrared) | Elysia Crampton |
| Bloody Mother Fucking Asshole | Martha Wainwright |
| The Prayer | Carmel |
| I Don't Feel Like Dancin' - Radio Edit | Scissor Sisters |
| The Streets Where I Belong | Annie |
| If You Knew - Live at Sin-é, New York, NY - July/August 1993 | Jeff Buckley |
| Medicine For Healthy People | Lim Giong |
| Don't Take My Soul | Jane Weaver |
| This Must Be the Place (Naive Melody) - 2005 Remaster | Talking Heads |
| Born This Way - The Country Road Version | Orville Peck |
| Baltimore | Nina Simone |
| Nature Boy | Nick Cave & the Bad Seeds |
| Good Fortune | PJ Harvey |
| Parallel Jalebi | Four Tet |
| Feeling Good | Nina Simone |
| Becuz | Sonic Youth |
| Nightclubbing | Grace Jones |
| CAMO | BoA |
| 砂之器 | Jer 柳應廷 |
| 無限未来 | Perfume |
| While You Dooooo (Extended) | Teebs |
| Bias | Floating Points |
| Inner City Blues (Make Me Wanna Holler) | Marvin Gaye |
| "You Should Be Dancing - Edit / From ""Saturday Night Fever"" Soundtrack" | Bee Gees |

**Table S3**

*Demographics of participants*

| Baseline characteristic | | All participants - questionnaires, eye-tracking or mobile EEG  (N=108) | | With eye-tracking data (N=45) | | With EEG data (N=34. Note: Demographics data was lost for 12 participants) | |
| --- | --- | --- | --- | --- | --- | --- | --- |
|  |  | M | SD | M | SD | M | SD |
| Age |  | 36.34 | 12.25 | 34.7 | 12.48 | 37.91 | 13.97 |
|  |  | % | | % | | % |  |
| Gender | Female | 61.11 |  | 60 |  | 52.94 |  |
|  | Male | 32.41 |  | 35.56 |  | 41.18 |  |
|  | Other | 55.56 |  | 4.44 |  | 5.88 |  |
|  | Prefer not to say | 0.93 |  | 0 |  | 0 |  |
| Ethnicity | Arab | 0.93 |  | 2.22 |  | 2.94 |  |
|  | Asian or Asian British | 12.96 |  | 13.33 |  | 11.76 |  |
|  | Black or Black British | 2.78 |  | 2.22 |  | 0 |  |
|  | Mixed | 3.71 |  | 4.44 |  | 2.94 |  |
|  | Other | 3.70 |  | 4.44 |  | 2.94 |  |
|  | Prefer not to say | 2.78 |  |  |  |  |  |
|  | White | 73.15 |  | 71.11 |  | 79.41 |  |
| Highest Educational Level | GCSEs or equivalent | 3.70 |  | 4.44 |  | 2.94 |  |
|  | A Levels or equivalent | 7.41 |  | 4.44 |  | 8.82 |  |
|  | University - Undergraduate Programme | 30.56 |  | 42.22 |  | 44.12 |  |
|  | University - Postgraduate Programme | 7.41 |  | 4.44 |  | 0 |  |
|  | Master's degree | 31.48 |  | 31.11 |  | 29.41 |  |
|  | Doctoral degree | 16.67 |  | 13.33 |  | 14.71 |  |

**Table S4**

*Participant sample sizes for eye-movement and EEG analyses after pre-processing*

| Stage | | Eye-tracking participants | | EEG participants | |
| --- | --- | --- | --- | --- | --- |
|  |  | Number | Analysis | Number | Analysis |
| Unique audience members equipped with the sensor | | 88 | Abstract | 77 | x |
| Recordings usable | | 83 | Before quality exclusions | 51 | Before quality exclusions |
| Data quality checks | After ICA rejection | x | x | 46 | EEG power spectrum analyses |
|  | With pre- and post-performance questionnaire data | 45 | In the Demographics Table S3. | x | x |
|  | With post-performance questionnaire fully completed, and fully attended individual performances | 40 | Correlation and linear mixed models, mediation analysis between ‘looking at people in the scene’ and pre- and post-performance ratings. | 34 | Correlation and linear mixed effect models between EEG power and post-performance ratings. In the Demographics Table S3. |
|  | Participants that had seen at least one group and one individual performance | x |  | 26 | Group vs. individual performance comparison and EEG power |
| EEG and eye trackers after data quality checks on both parts | | 40 | | | EEG power and ‘looking at people’ in the scene correlations. |

**Table S5**

*EEG recording characteristics and independent component rejection by participant (N = 46)*

| **Participant ID** | **Total recording time (min)** | **Mean music-section time (min)** | **Mean no-music-section time (min)** | **No. of performers watched** | **No. of components rejected** |
| --- | --- | --- | --- | --- | --- |
| sub-03 | 57.26 | 3.61 | 3.08 | 4 | 23 |
| sub-13 | 73.41 | 5.25 | 3.08 | 4 | 22 |
| sub-24 | 12.34 | 3.45 | 2.72 | 1 | 25 |
| sub-32 | 86.31 | 3.81 | 2.65 | 5 | 19 |
| sub-37 | 31.71 | 4.86 | 3.61 | 2 | 21 |
| sub-47 | 69.30 | 4.49 | 4.12 | 4 | 25 |
| sub-49 | 45.66 | 4.10 | 2.94 | 2 | 21 |
| sub-55 | 102.01 | 3.98 | 4.03 | 5 | 24 |
| sub-56 | 66.25 | 3.28 | 2.81 | 5 | 17 |
| sub-57 | 66.25 | 3.28 | 2.81 | 5 | 24 |
| sub-58 | 77.64 | 4.07 | 2.48 | 5 | 21 |
| sub-61 | 64.32 | 3.90 | 3.33 | 4 | 21 |
| sub-62 | 75.38 | 4.19 | 2.34 | 4 | 27 |
| sub-64 | 73.15 | 4.44 | 4.12 | 4 | 25 |
| sub-69 | 85.67 | 4.18 | 3.42 | 4 | 20 |
| sub-76 | 67.78 | 4.18 | 4.93 | 3 | 20 |
| sub-83 | 85.52 | 4.18 | 3.42 | 4 | 22 |
| sub-85 | 55.13 | 4.58 | 3.84 | 3 | 23 |
| sub-87 | 114.99 | 3.63 | 3.73 | 6 | 16 |
| sub-90 | 32.62 | 3.64 | 5.43 | 2 | 21 |
| sub-95 | 72.21 | 4.33 | 3.10 | 4 | 18 |
| sub-96 | 70.48 | 4.18 | 3.49 | 3 | 23 |
| sub-102 | 110.56 | 3.63 | 3.82 | 6 | 22 |
| sub-104 | 68.42 | 5.41 | 3.08 | 3 | 17 |
| sub-107 | 101.34 | 3.61 | 3.14 | 6 | 24 |
| sub-111 | 50.74 | 3.49 | 3.49 | 3 | 18 |
| sub-112 | 68.36 | 3.07 | 3.23 | 5 | 24 |
| sub-115 | 76.43 | 5.27 | 3.44 | 4 | 22 |
| sub-126 | 84.37 | 3.84 | 3.53 | 5 | 24 |
| sub-128 | 36.98 | 3.52 | 4.57 | 3 | 22 |
| sub-131 | 50.74 | 3.49 | 3.49 | 3 | 19 |
| sub-133 | 93.32 | 3.57 | 3.57 | 5 | 23 |
| sub-134 | 77.78 | 4.18 | 3.78 | 5 | 27 |
| sub-136 | 35.51 | 4.85 | 3.07 | 2 | 22 |
| sub-137 | 23.74 | 3.99 | 3.39 | 2 | 23 |
| sub-139 | 57.17 | 3.49 | 3.85 | 4 | 23 |
| sub-140 | 62.22 | 3.49 | 4.22 | 3 | 22 |
| sub-141 | 69.94 | 3.77 | 3.91 | 4 | 21 |
| sub-142 | 69.94 | 3.77 | 3.91 | 4 | 20 |
| sub-148 | 50.62 | 4.31 | 3.47 | 3 | 20 |
| sub-149 | 105.74 | 3.60 | 3.76 | 6 | 21 |
| sub-159 | 72.83 | 3.99 | 2.78 | 5 | 23 |
| sub-169 | 102.18 | 3.98 | 4.03 | 5 | 21 |
| sub-170 | 18.21 | 4.30 | 2.94 | 2 | 17 |
| sub-172 | 87.19 | 3.46 | 3.21 | 5 | 22 |
| sub-187 | 52.05 | 4.58 | 3.84 | 3 | 25 |
| Average | 67.65 | 4.01 | 3.50 | 3.89 | 21.74 |

*Note.* Participant identifiers correspond to BIDS subject labels in the associated OpenNeuro dataset (doi:10.18112/openneuro.ds007802) and are not linked to participant identifiers used elsewhere in this manuscript or its supplementary materials. Full lists of rejected independent components per participant are available in the EEG preprocessing pipeline output on OSF (see Data Availability Statement).

**Table S6**

*Spearman correlations between ratings and percentage of looking at the performer*

| Variable | Fam | Foc | FC | HC | FN | FP | HN | HP | Look |
| --- | --- | --- | --- | --- | --- | --- | --- | --- | --- |
| Connection | 0.18 | **0.73**  **<0.001** | 0.03 | 0.11 | -0.00 | **0.31**  **0.002** | 0.06 | 0.18 | 0.13 |
| Familiarity (Fam) | _ | **0.28**  **0.006** | -0.05 | -0.04 | -0.19 | 0.01 | -0.01 | 0.11 | 0.09 |
| Focus (Foc) |  | _ | -0.09 | 0.02 | -0.17 | 0.17 | -0.01 | 0.16 | **0.35**  **<0.001** |
| Feeling count (FC) |  |  | _ | **0.53**  **<0.001** | **0.26**  **0.009** | **0.45**  **<0.001** | 0.13 | **0.22**  **0.035** | -0.08 |
| Happened count (HC) |  |  |  | _ | 0.14 | **0.32**  **0.001** | **0.26**  **0.009** | **0.44**  **<0.001** | -0.13 |
| Feeling negative (FN) |  |  |  |  | _ | -0.03 | -0.02 | -0.08 | -0.08 |
| Feeling positive (FP) |  |  |  |  |  | _ | 0.13 | **0.31**  **0.002** | 0.18 |
| Happened negative (HN) |  |  |  |  |  |  | _ | 0.13 | -0.03 |
| Happened positive (HP) |  |  |  |  |  |  |  | _ | 0.01 |
| Looking at others (Look) |  |  |  |  |  |  |  |  | _ |

*Note*. In each case, the number on the first line indicates the strength of the correlation and the one on the second line reports the p-value, after application of the Benjamini-Hochberg correction. Significant values are in bold (p < 0.05).

**Table S7**

*Spearman correlations between personal characteristics and all the other variables*

| Variable | MWS | MWD | Emp | Agr | Cons | Ext | Neu | Ope | ODE | PDE |
| --- | --- | --- | --- | --- | --- | --- | --- | --- | --- | --- |
| Look at others (Look) | **-0.3**  **0.003** | **-0.3**  **0.003** | -0.09 | -0.13 | -0.15 | 0.1 | **0.3**  **0.003** | -0.17 | **-0.45 <0.001** | -0.11 |
| Connection | -0.15 | -0.14 | 0.05 | 0.04 | -0.07 | -0.1 | -0.02 | -0.01 | -0.08 | 0.00 |
| Familiarity (Fam) | -0.10 | **-0.22 0.035** | **-0.24 0.021** | -0.02 | -0.13 | -0.11 | 0.14 | 0.1 | 0.11 | 0.07 |
| Focus (Foc) | **-0.26 0.012** | **-0.26 0.011** | -0.1 | -0.14 | -0.2 | 0.05 | **0.24 0.02** | -0.08 | **-0.24 0.024** | 0.07 |
| Feeling count (FC) | 0.12 | **-0.27 0.009** | **0.32 0.001** | 0.00 | 0.13 | 0.11 | -0.2 | -0.17 | -0.09 | -0.06 |
| Happened count (HC) | 0.01 | -0.15 | **0.25 0.017** | -0.00 | 0.13 | 0.02 | -0.21 | -0.03 | 0.02 | 0.17 |
| Feeling negative (FN) | 0.21 | -0.05 | 0.10 | -0.19 | -0.07 | -0.13 | -0.06 | -0.05 | 0.02 | **-0.23 0.031** |
| Feeling positive (FP) | -0.18 | -0.1 | 0.08 | 0.11 | 0.04 | -0.03 | 0.02 | 0.01 | -0.19 | 0.05 |
| Happened negative (HN) | -0.09 | -0.12 | -0.04 | -0.06 | -0.14 | -0.09 | 0.1 | -0.02 | 0.01 | 0.11 |
| Happened positive (HP) | -0.11 | -0.04 | 0.06 | 0.14 | **0.25 0.015** | 0.16 | -0.03 | 0.07 | -0.09 | 0.2 |
| Mind wandering spontaneous (MWS) | _ | 0.18 | **0.51 <0.001** | -0.15 | 0.07 | -0.12 | -0.12 | 0.14 | -0.07 | **-0.54 <0.001** |
| Mind wandering deliberate (MWD) |  | _ | 0.04 | 0.16 | **0.39**  **<0.001** | -0.04 | **-0.29 0.004** | **0.39 <0.001** | **0.3 0.003** | 0.12 |
| Empathy (Emp) |  |  | _ | -0.11 | 0.16 | 0.08 | -0.08 | **-0.3 0.003** | **-0.32 0.001** | **-0.42 <0.001** |
| Agreeableness (Agr) |  |  |  | _ | **0.41 <0.001** | **0.22 0.035** | **-0.52 <0.001** | **0.22 0.037** | 0.15 | 0.18 |
| Conscientiousness (Cons) |  |  |  |  | _ | **0.28 0.005** | **-0.54 <0.001** | **0.21 0.047** | 0.2 | 0.18 |
| Extraversion (Ext) |  |  |  |  |  | _ | -0.03 | -0.04 | -0.12 | 0.1 |
| Neuroticism (Neu) |  |  |  |  |  |  | _ | -0.05 | **-0.33 0.001** | -0.11 |
| Openness (Ope) |  |  |  |  |  |  |  | _ | **0.5 <0.001** | **0.29 0.003** |
| Observational Dance Experience (ODE) |  |  |  |  |  |  |  |  | _ | **0.56 <0.001** |
| Participatory Dance Experience (PDE) |  |  |  |  |  |  |  |  |  | _ |

*Note.* In each case, the number on the first line indicates the strength of the correlation and the one on the second line reports the p-value, after application of the Benjamini-Hochberg correction. Significant values are in bold (p < 0.05).
